# Appropriate medication use in Dutch terminal care: study protocol of a multicentre stepped-wedge cluster randomized controlled trial (the AMUSE study)

**DOI:** 10.1186/s12904-023-01334-x

**Published:** 2024-01-03

**Authors:** M.A.M. van Hylckama Vlieg, I.E. Pot, H.P.J. Visser, M.A.C. Jong, M.J.D.L. van der Vorst, B.J. van Mastrigt, J.N.A. Kiers, P.P.P.H. van den Homberg, M.F. Thijs-Visser, E. Oomen-de Hoop, A. van der Heide, P.H.M. van der Kuy, C.C.D. van der Rijt, E.C.T. Geijteman

**Affiliations:** 1 Department of Medical Oncology, Erasmus MC Cancer Institute, Rotterdam, The Netherlands; 2 Department of Internal Medicine, Noordwest Ziekenhuis, Alkmaar, The Netherlands; 3 Department of Internal Medicine, Center for Supportive and Palliative Care, Rijnstate Hospital, Arnhem, The Netherlands; 4 Laurens Cadenza Zuid, Rotterdam, The Netherlands; 5 Family Medicine Network, Nijmegen, The Netherlands; 6 Gezondheidscentrum Krimpen, Krimpen aan den IJssel, The Netherlands; 7 Department of Medical Oncology, Ikazia Hospital, Rotterdam, The Netherlands; 8 Department of Public Health, Erasmus MC, University Medical Center Rotterdam, Rotterdam, The Netherlands; 9 Department of Hospital Pharmacy, Erasmus MC, University Medical Center Rotterdam, Rotterdam, The Netherlands

**Keywords:** Potentially inappropriate medications (PIMs), Clinical decision support systems, Drug therapy, Deprescribing, Quality of life, Stepped-wedge cluster randomized controlled trial, Palliative care

## Abstract

**Background:**

Polypharmacy is common among patients with a limited life expectancy, even shortly before death. This is partly inevitable, because these patients often have multiple symptoms which need to be alleviated. However, the use of potentially inappropriate medications (PIMs) in these patients is also common. Although patients and relatives are often willing to deprescribe medication, physicians are sometimes reluctant due to the lack of evidence on appropriate medication management for patients in the last phase of life. The aim of the AMUSE study is to investigate whether the use of CDSS-OPTIMED, a software program that gives weekly personalized medication recommendations to attending physicians of patients with a limited life expectancy, improves patients’ quality of life.

**Methods:**

A multicentre stepped-wedge cluster randomized controlled trial will be conducted among patients with a life expectancy of three months or less. The stepped-wedge cluster design, where the clusters are the different study sites, involves sequential crossover of clusters from control to intervention until all clusters are exposed. In total, seven sites (4 hospitals, 2 general practices and 1 hospice from the Netherlands) will participate in this study. During the control period, patients will receive ‘care as usual’. During the intervention period, CDSS-OPTIMED will be activated. CDSS-OPTIMED is a validated software program that analyses the use of medication based on a specific set of clinical rules for patients with a limited life expectancy. The software program will provide the attending physicians with weekly personalized medication recommendations. The primary outcome of this study is patients’ quality of life two weeks after baseline assessment as measured by the EORTC QLQ-C15-PAL questionnaire, quality of life question.

**Discussion:**

This will be the first study investigating the effect of weekly personalized medication recommendations to attending physicians on the quality of life of patients with a limited life expectancy. We hypothesize that the CDSS-OPTIMED intervention could lead to improved quality of life in patients with a life expectancy of three months or less.

**Trial registration:**

This trial is registered at ClinicalTrials.gov (NCT05351281, Registration Date: April 11, 2022).

**Supplementary Information:**

The online version contains supplementary material available at 10.1186/s12904-023-01334-x.

## Background

Polypharmacy is common among patients with a limited life expectancy, even shortly before death [1–7]. This is partly inevitable, because these patients often have multiple symptoms which need to be alleviated [8, 9]. However, the benefits of using several other types of medications at the end of life are debatable for a number of reasons. Firstly, some medications are not effective anymore, given the limited life expectancy of the patient. This mainly applies for preventive medications, such as statins, which have benefits which take months to years to accrue [10]. Secondly, changing treatment objectives at the end of life may directly affect medication strategies. An example is the treatment of diabetes mellitus. In case of a limited life expectancy it is better to raise the upper level of a patient’s glycated haemoglobin (A1C) above 7%, to prevent short-term complications, such as hypoglycaemia [11]. Thirdly, organ functions may undergo changes when death is nearing [12]. For instance, blood pressure may drop in the last weeks of life, which makes anti-hypertensive medications no longer necessary and even potentially harmful [12]. When a patient has a limited life expectancy, physicians should therefore reconsider the use of potentially inappropriate medications (PIMs) [13–15]. PIMs can be defined as medications that have a greater potential risk than expected clinical benefit and, therefore, should be avoided [16].

Polypharmacy has been associated with an increased risk of adverse events, a higher symptom burden, and a lower quality of life [15, 17]. There is growing awareness that the use of medications needs careful consideration and that PIMs should be discontinued whenever possible [14, 18, 19]. As early as 2015, a call was issued for more research on deprescribing medications aimed at prevention or treatment of diseases which are irrelevant in the light of patients’ limited life expectancy [18]. Patients and relatives are often willing to deprescribe medications if their physician informed them that it was possible [20, 21]. However, physicians are sometimes reluctant for various reasons [20–22]. In an interview and questionnaire study of reasons why medication review at the end of life is rare, we found that physicians seem to be concerned about potential negative medical consequences of discontinuing medication, such as the occurrence of symptomatic hyperglycaemia when discontinuing glucose lowering medications, and are in need of clinical guidelines for deprescribing [20, 23–25]. In addition, limited awareness seems an important reason why medications are continued until the very end of life [20].

Until now, a limited number of ‘deprescribing trials’ have been conducted, which are studies of the effects and safety of discontinuing medication in the last phase of life [14, 26]. Moreover, only one randomized controlled trial has explored the discontinuation of medication in patients with a limited life expectancy [10]. This trial evaluated the safety of discontinuing statins for patients without recent cardiovascular events whose estimated life expectancy was between one month and one year. The results demonstrated that discontinuation of statins in this patient population was safe, and even associated with improved quality of life [10].

To support physicians in deprescribing medication in patients in the last phase of life, we have designed a study to evaluate the effect of personalized medication management recommendations on quality of life in patients in the last phase of life. The recommendations are provided by a clinical decision support system (CDSS) and concern medications which may be discontinued in patients who have a life expectancy of three months or less (CDSS-OPTIMED). The recommendations are based on scientific literature, currently available guidelines and pharmacological insights. We hypothesize that the CDSS-OPTIMED intervention could lead to improved quality of life in patients with a life expectancy of three months or less. In addition, we expect that the intervention will lead to improved medication management.

## Methods

### Overall aim

The overall aim of this study is to investigate whether the use of CDSS-OPTIMED, a software program that provides weekly personalized medication recommendations to attending physicians of patients with an estimated life expectancy of three months or less, improves patients’ quality of life.

### Setting

This study will be conducted in seven different study sites throughout the Netherlands. Patients will be recruited in four hospitals (Internal Medicine and Oncology departments), two general practices, and one hospice. Both patients from the inpatient clinic and outpatient clinic from one academic and three non-academic hospitals will be recruited.

### Study design

**Fig. 1:**
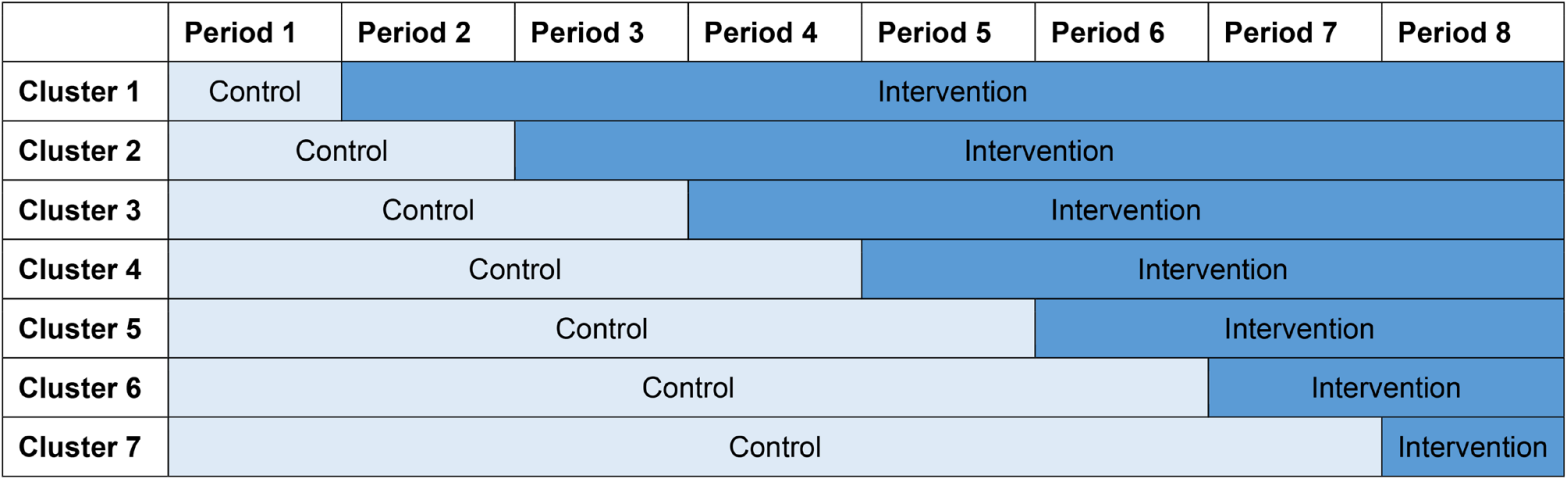
Schematic illustration of the stepped-wedge cluster randomized controlled study design. Every period is 12 weeks

The AMUSE study is a multicentre stepped-wedge cluster randomized controlled trial that evaluates the effect of weekly personalized medication recommendations provided by CDSS-OPTIMED. The stepped-wedge design will involve random and sequential crossover of clusters, the different study sites being the clusters, from the control to the intervention group, until all clusters are exposed. A schematic illustration of the study method is highlighted in Fig. 1. All clusters will start as control sites, where care is provided as usual. Every twelve weeks, one study site will crossover from control to intervention group. Since seven study sites will be included and will switch from control to intervention with one site per step, seven steps are included, resulting in eight observation periods in 96 weeks. Sites will crossover from control to the intervention group in a random order. Within two weeks before and ultimately two weeks after starting the crossover, physicians will be trained to use CDSS-OPTIMED. Within these four weeks of training there will be a period of non-inclusion. In the intervention group, the attending physicians will receive weekly personalized medication recommendations.

Study outcomes, paper or electronically sent questionnaires, will be collected upon patients’ inclusion in the study, at day 7, 14, 21, 28, and then every 28 days until death or as long the patient is able to fill in the questionnaire, with a maximum of 24 weeks.

The study is designed in accordance with the Declaration of Helsinki and the principles of Good Clinical Practice (GCP) set by the International Conference of Harmonization (ICH) [27, 28]. It has been approved by the Medical Ethical Research Committee of the Erasmus MC, University Medical Center Rotterdam. Patients provide written informed consent before participation. The study is registered in ClinicalTrials.gov, registration number NCT05351281, Registration Date: April 11, 2022. Patient recruitment and data collection have started in April 2022.

### Study population

We will include patients with a life expectancy of at least two weeks and at most three months, as estimated by the attending physician. Additional inclusion criteria are that the patient is ≥ 18 years old, provides informed consent, and is aware that recovering from his/her disease is unlikely, as assessed by the attending physician. Exclusion criterion for participation includes incapability of filling in a questionnaire with questions in Dutch.

### Randomization, blinding and treatment allocation

Sites will crossover from control group to the intervention group in a random order. A randomization list will be generated by the statistician before the start of the study that takes into account the type of site (hospitals versus general practices/hospice) as these differ in size. Within two weeks before the crossover, the local investigator of the study site will be notified of the crossover date. Given the nature of the intervention, blinding is not possible.

### Intervention

After the crossover from control to intervention group, the attending physician will receive personalized medication recommendations provided by CDSS-OPTIMED as soon as possible, within the first week after the inclusion of the patient, and thereafter each week until the patient’s death. CDSS is a Clinical Decision Support System that was developed to guide physicians in medication prescription and deprescription in elderly patients using numerous medications [29, 30]. For the current study, we have adapted the CDSS to CDSS-OPTIMED that is aimed at optimizing medication deprescription in patients with a limited life expectancy. CDSS-OPTIMED is a validated software program that will analyse the use of medication based on a specific set of clinical rules for patients with a limited life expectancy. The software program will provide the attending physicians with weekly personalized medication recommendations about deprescription. Medication currently used by the patient will be obtained through the LSP (Landelijk Schakelpunt – or National Exchange Point) and will be sent to the software program. All medication recommendations are based on currently available guidelines, pharmacological insights, and evidence from scientific literature [10, 31–34]. The clinical rules, which will be specifically added to the CDSS-OPTIMED, are based on a recently performed Delphi study among an international panel of 47 experts (submitted). See Supplementary S1 and S2 for a more detailed explanation of this Delphi study and the resulting medication recommendations. CDSS-OPTIMED will give a detailed guidance on which drugs, in which order, and for which indications can be deprescribed.

In order to guarantee adequate use of the CDSS-OPTIMED and to stimulate appropriate communication with the patient and relatives about the alerts, physicians in each site will be trained. This training includes advice on how to communicate with patients and their families about medication management in the last phase of life. The attending physician will remain fully responsible for any medication deprescription and will be free to follow or ignore the medication recommendation, based on their expertise and the individual patient’s situation. If needed, the attending physician can discuss the medication recommendation with the pharmacist before discussing this recommendation with the patient and/or the relative. The ultimate decisions and actions taken by the physician will be registered in CDSS-OPTIMED and patient’s medical record.

### Study measurements

Demographic and baseline data will be extracted from the medical record and the patient’s baseline questionnaire. This includes age, gender, current living status, highest completed level of education, performance status, main diagnosis, comorbidities, and medication use.

The primary outcome of this study is patients’ quality of life two weeks after baseline assessment as measured by the EORTC QLQ-C15-PAL questionnaire, quality of life question. The EORTC QLQ-C15-PAL questionnaire is a patient-reported questionnaire to assess health-related quality of life, and is an abbreviated 15-item version of the EORTC QLQ-C30 questionnaire developed to be used in a palliative care setting [35]. In this study, we will only use the quality of life question of the EORTC QLQ-C15-PAL questionnaire: *“How would you rate your overall quality of life during the past week?”* rated on a scale of 1 (very poor) to 7 (excellent). Scores will be rescaled to a scale from 0 to 100 [36].

Secondary outcomes are:

- Patients’ quality of life at baseline, day 7, 21, 28, and then every 28 days until end of study, as measured by the EORTC QLQ-C15-PAL questionnaire, quality of life question;
- Symptoms and the occurrence of potential side effects of continuing or discontinuing medication at baseline, day 7, 21, 28, and then every 28 days until end of study, as assessed by the Utrecht Symptom Diary (USD), which is based on the Edmonton Symptom Assessment Scale (ESAS) and is frequently used in palliative care;
- Blood pressure and glucose level, in case of use of antihypertensives and/or antidiabetics, at baseline, day 7, 14, 21, 28, and then every 28 days until end of study;
- Thrombo-embolic and bleeding events;
- Time spent on discussing the medication with the patient, as registered on a time scale (0–5 min, 6–10, 11–15 min, 16–20 min, and > 21 min);
- Medication used until death, as derived from patients’ medical records and the pharmacist’s information system;
- Survival from the moment of inclusion;
- Health care costs; to conduct a health economic analysis.

### Sample size calculation

The primary endpoint on which the sample size calculation is based is patients’ quality of life two weeks after baseline assessment as measured by the EORTC QLQ-C15 PAL questionnaire, quality of life question. The calculation needs to take into account both the clustered nature as well as the stepped wedge design of the study. Since 7 clusters will be included and will switch from control to intervention one-by-one, 7 steps will be present in the design, resulting in 8 observation periods. The intra-cluster correlation (ICC) is assumed to be 0.05. Based on unpublished own research, it is assumed that the standard deviation of quality of life measured by the EORTC QLQ-C15-PAL questionnaire is 23 (score will be rescaled to a scale from 0 to 100) and the difference to be detected should lie between 10 and 15 points. The desired power level is 80% and a two-sided alpha of 5% is used. Given expected numbers of available patients per cluster per month, the average number of patients that will be recruited per observation period per cluster will be 4.5. If the average number is 4, a total number of 224 patients will be included, whereas a total of 280 patients will be included when the average number is 5. Given these sample sizes, differences can be shown with 80% power if they are at least 13.03 points and 11.84 points, respectively [37, 38]. Since the average number of patients per cluster per observation period lies in between 4 and 5, it is expected that a total of 250 patients will be included and a clinically relevant effect can be shown.

### Statistical analysis

All data will be coded and collected in a secure web browser clinical data management platform (ALEA Clinical). All analyses will be performed using the latest version of IBM SPSS Statistics and R [39], will be two sided and considered significant if p < 0.05. Multivariate Imputation by Chained Equations (MICE) will be used to handle missing data where possible. Descriptive statistics will be generated to summarize patients’ demographics and medical disease characteristics by study site and by intervention (i.e. without vs. with use of CDSS-OPTIMED).

As assessed with an intention-to-treat approach, we will compare patients’ quality of life between patients who were enrolled in the study prior to the use of the CDSS-OPTIMED and patients who were enrolled after implementation of the CDSS-OPTIMED. In a multilevel multivariable regression analysis, with quality of life as the dependent variable, we will take into account the stepped wedge cluster randomized design of the study, and potential baseline differences (including baseline quality of life) between patients in the intervention and control group.

The following secondary analyses will be performed. Firstly, symptom scores at day 7, 14, 21, 28 and then every 28 days until death after baseline assessment will be compared between patients who were enrolled in the study prior to the use of the CDSS-OPTIMED and patients who were enrolled after implementation of the CDSS-OPTIMED, using a multilevel multivariable regression analysis and an intention to treat approach, similar to the analysis of the primary outcome. Secondly, similar analyses will be performed to compare patients’ quality of life at day 7, 21, 28, and then every 28 days until death. Thirdly, cox regression analysis will be used to compare overall survival. Fourthly, the use of medication two weeks after baseline assessment and used medications and interventions from baseline until death will be compared between the respective groups of patients using parametric or non-parametric methods, where appropriate. Furthermore, a cost-effectiveness analysis, using a within-trial incremental cost-effectiveness analysis, will be performed to compare the control group (‘care as usual’) with the intervention group (the use of CDSS-OPTIMED).

## Discussion

The AMUSE study is a multicentre stepped-wedge cluster randomized controlled trial that examines whether the use of CDSS-OPTIMED, a software program that gives weekly personalized medication recommendations to attending physicians of patients with a life expectancy of three months or less, improves patients’ quality of life.

This is the first study investigating whether carefully reviewing medication use of patients with a limited life expectancy, by means of weekly personalized medication recommendations, will increase patients’ quality of life. Until now, the exact consequences of carefully reviewing medication use in the last phase of life are lacking.

There is growing awareness that the use of medication in patients with a limited life expectancy needs careful consideration and that PIMs should be discontinued whenever possible [14, 18, 19]. To give physicians more guidance on medication deprescription in the last phase of life, different medication deprescribing tools and guidelines were developed for the geriatric population [29, 40, 41], and for patients with advanced cancer [7, 34, 42–44]. However, none of these tools or guidelines are aimed at the general population with a limited life expectancy. Improving medication deprescription in patients with a limited life expectancy requires an approach that is specifically designed and validated for this population. Recently, a Delphi study analysed the existing literature to develop recommendations on deprescribing medications for patients with a life expectancy of six months maximum. Our study integrated these recommendations into the software program CDSS-OPTIMED.

By using the validated software program CDSS-OPTIMED, medication reviews and therefore medication recommendations are provided in a standardized automatically way, which will result in more well-considered prescription [45]. In addition, the software program is expected to be user-friendly and is expected to save time compared to manual medication review, and will therefore improve medication review effectiveness [46]. Another advantage of using a validated software program is that the medication recommendations, based on the analysis of patient’s medication, will be given automatically on a weekly basis. Since limited awareness of proactively deprescribing medication among physicians is an important reason why medication is continued [20], we expect that recommendations given on a regular basis will create lasting awareness among physicians and provide them with continuous practical support for effective medication management.

This study has a multicentre design, to be conducted in seven different study sites across the Netherlands: one inpatient hospice facility, two general practices and four hospitals. By including patients with a limited life expectancy from multiple clinical sites and including patients with different main diagnoses and comorbidities, we expect that our study findings can ultimately be widely implemented.

We also expect to encounter several challenges in the AMUSE study. Firstly, the main challenge will be recruiting patients with a limited life expectancy for research. Patients with a limited life expectancy are considered vulnerable and fragile due to their illness, and may experience fluctuating symptoms and levels of suffering across their disease trajectory [47]. In addition, relatives or healthcare professionals may be hesitant to grant researchers access to incurably ill patients, due to concerns about burdening or distressing them, a phenomenon referred to as ‘gatekeeping’ [48]. However, multiple studies have shown that patients who are receiving palliative care are willing to participate in medical research and may benefit from their participation [47, 49, 50]. Nevertheless, it is important to reduce the burden during the study for patients with a limited life expectancy [51]. To reduce such burden, we will use short questionnaires for patients to complete, and we will give patients the opportunity to choose how the questionnaires will be completed (digitally or on paper). To minimize problems relating to the number of included patients, we have involved multiple clinical sites and planned for modest numbers of participants per site.

Secondly, another challenge of the study could be the high drop-out of patients as a result of patient’s death, gatekeeping [48], or patient’s deteriorating health condition [47]. Consequently, patients may not reach our primary endpoint two weeks after the baseline assessment and may not be able to complete the follow-up period. To minimize the risk of drop-out, we will maintain frequent contact with the patient during the study if the patient has given permission for this. In addition, we strive to minimize the drop-out by reducing the burden for patients by using only short questionnaires.

Lastly, another challenge of this study could be the methodological complexities of the stepped wedge cluster randomized controlled trial design. This is a novel study design that is increasingly being used in the evaluation of service innovations in healthcare organisations [38, 52]. We have chosen this study design because the CDSS-OPTIMED intervention cannot be implemented all at once but must be rolled out sequentially. However, the stepped wedge design has also some methodological complexities, including the possibility of within cluster contamination, and the possibility of time varying treatment effects [38]. Statistical analysis and simple size calculation will account for the confounding effect of time. In addition, the statistical analysis will take into account both the clustered nature as well as the stepped wedge design of the study. In developing this study, we have tried to avoid within-cluster contamination, because participating patients cannot be exposed to both the control group and the intervention group, and we have built transition periods into the design.

## Conclusions

The AMUSE study will examine whether the use of CDSS-OPTIMED, a software program that gives weekly personalized medication recommendations to attending physicians of patients with an estimated life expectancy of three months or less, improves patients’ quality of life. We hypothesize that greater attention to polypharmacy, by using CDSS-OPTIMED, could lead to improved quality of life in patients with a life expectancy of three months or less. In addition, we expect that the intervention will lead to improved medication management. If this study has its hypothesized positive outcome, the results of this study can be used to develop practical guidelines and a standardized method to help physicians with deprescribing medication.

### Electronic supplementary material

Below is the link to the electronic supplementary material.

**Supplementary Material 1:** Supplementary S1 - Detailed explanation of the Delphi Study

## Data Availability

No datasets were generated or analysed during the current study.

## Abbreviations

CDSS-OPTIMED: Clinical Decision Support System for patients with a life expectancy of less than three months
CONSORT: Consolidated Standards of Reporting Trials
EORTC: European Organization for Research and Treatment of Cancer
PIM: Potentially Inappropriate Medication
QLQ-C15-PAL: Quality of Life Questionnaire Core 15 Palliative Care
QLQ-C30: Quality of Life Questionnaire Core 30
