# Supplementary material for: Appropriate medication use in Dutch terminal care: study protocol of a multicentre stepped-wedge cluster randomized controlled trial (the AMUSE study)
Source: BMC Palliat Care. 2024 Jan 3;23:6. doi: 10.1186/s12904-023-01334-x (PMC10762916; doi:10.1186/s12904-023-01334-x)
Supplement: Supplementary file 1 — Supplementary Material 1: Supplementary S1 - Detailed explanation of the Delphi Study [file 12904_2023_1334_MOESM1_ESM.docx]

**Supplementary material**

**Supplementary S1 - Detailed explanation of the Delphi Study**

Recently, a Delphi study analysed the existing literature to develop recommendations on deprescribing medications for patients with a life expectancy of six months maximum. An international and multidisciplinary panel of 47 experts in palliative care and other medical disciplines were asked to give their advice in this international Delphi study. A list of 42 recommendations regarding the consideration of deprescription of various medications were formulated. In two Delphi rounds, participating experts were asked to rate their agreement with each recommendation. Consensus was reached, i.e. recommendations were accepted, if at least 70% of the participating experts (strongly) agreed, the interquartile range (IQR) was one or less, and if less than 10% of the experts strongly disagreed. In most cases (86%), consensus was reached for deprescribing medications for patients with a life expectancy of 6 months or less. The highest level of consensus for medications considered to deprescibe was reached for: diuretics in case of decreasing fluid intake or increasing fluid loss, lipid modifying agents if prescribed for primary prevention, and vitamin K antagonists and direct oral anticoagulants in case of high risk for bleeding.

This study has currently been submitted to a journal, as a result referring to this manuscript is not yet possible.

**Supplementary S2 - Delphi-study: Recommendations about deprescribing medication**

| **Life-expectancy: Six months or less** | | | | | | | | | | | | |
| --- | --- | --- | --- | --- | --- | --- | --- | --- | --- | --- | --- | --- |
| **Medication Class** | **Medication** | | **ATC code** | **Recommendation** | | | | | **% Agreement** | **IQR** | **% Strongly disagree** |  |
| Cardiovascular medication | ACE-inhibitor / Angiotensin II receptor blockers  Beta-blocking agents  Centrally acting antiadrenergic agents  Alpha-adrenoreceptor antagonists  Calcium Channel blockers  Diuretics  Organic Nitrates  Digoxin  Lipid modifying agents | | C09  C07  C02A  C02CA  C08  C03  C01DA  C01AA  C10 | Consider deprescribing ACE-inhibitor / Angiotensin-II-receptor blocker if prescribed for primary prevention of diabetic nephropathy  Consider tapering and if possible stopping beta blocking agents if prescribed for mild-moderate hypertension (systolic blood pressure ≤ 179, diastolic blood pressure ≤ 110 mmHg i.e. stage 1-2-3 hypertension)  Consider tapering and if possible stopping beta blocking agents in case of heart failure (NYHA class 1 to 4) without atrial fibrillation in combination with bradycardia (<50 beats/min)  Consider deprescribing centrally acting antiadrenergic agents if prescribed for hypertension (systolic blood pressure < 179, diastolic blood pressure < 110 mmHg i.e. stage 1-2-3 hypertension)  Consider deprescribing alpha-adrenoreceptor antagonists (prazosin, doxazosin, urapidil, terazosin) if prescribed for hypertension (all stages, i.e. systolic blood pressure >140 mmHg, diastolic blood pressure >90 mmHg)  Consider deprescribing calcium channel blockers if prescribed for mild-moderate hypertension (systolic blood pressure < 179, diastolic blood pressure < 110 mmHg i.e. stage 1-2-3 hypertension)  Consider deprescribing diuretics in case of decreasing fluid intake or increasing fluid loss (diarrhea, vomiting, excessive sweating)  Consider deprescribing organic nitrates if prescribed for asymptomatic angina (angina complaints in past history without recent (<1 yr) complaints)  Consider deprescribing digoxin if prescribed for asymptomatic atrial fibrillation (atrial fibrillation in past history without complaints now)  Consider deprescribing lipid modifying agents if prescribed for primary prevention (hypercholesterolemia) of cardiovascular disease  Consider deprescribing lipid modifying agents if prescribed for secondary prevention of cardiovascular disease (> 12 months since last incident, complaints or intervention such as stenting) | | | | | 93,6%  90,0%  83,7%  88,4%  90,9%  77,8%  100%  88,6%  76,2%  100%  97,9% | 1  1  1  1  1  1  0  1  1  0  1 | 0%  2,3%  2,3%  2,3%  2,3%  2,2%  0%  0%  0%  0%  0% |  |
| Antiplatelet therapy | Platelet aggregation inhibitor | | B01AC | Consider deprescribing platelet aggregation inhibitor (e.g. aspirin > 100 milligram per day, P2Y12-inhibitors) if prescribed for primary prevention of cardiovascular accident  Consider deprescribing platelet aggregation inhibitor (e.g. aspirin > 100 milligram per day, P2Y12-inhibitors) in case of an increased bleeding risk and if prescribed for secondary prevention of cardiovascular accident (e.g. > 12 months after incidents, complaints or interventions such as vascular stenting)  Consider deprescribing platelet aggregation inhibitor (e.g. aspirin, P2Y12-inhibitors) in case of a high risk at bleeding: - uncontrolled hypertension (systolic pressure >179 mmHg) - uncontrolled bleeding - relevant recent spontaneous bleeding - clinically relevant bleeding: medical intervention or (prolonged) hospital admission, unscheduled contact physician, discomfort, impairment of activities, multiple source bleeding, hemoglobin drop > 3 g/dL. - HASBLED >3 - kidney of liver failure:  Consider deprescribing platelet aggregation inhibitor (e.g. aspirin, P2Y12-inhibitors) in case of thrombocytopenia (<150000 platelets per microliter of blood) | | | | | 87,2%  88,4%  97,8%  84,1% | 1  1  0  1 | 0%  0%  0%  2,3% |  |
| Anticoagulants | Vitamin K antagonist  Heparins  Direct Oral Anticoagulants (DOAC) | | B01AA  B01AB  B01AF | Consider deprescribing vitamin K antagonist (acenocoumarol, fenprocoumon, warfarin) if prescribed for uncomplicated deep venous thrombosis or uncomplicated pulmonary embolism >6 months ago in patients without cancer  Consider deprescribing vitamin K antagonist (acenocoumarol, fenprocoumon, warfarin) in case of high risk at bleeding: - uncontrolled hypertension (systolic pressure >179 mmHg) - uncontrolled bleeding - relevant recent spontaneous bleeding - clinically relevant bleeding: medical intervention or (prolonged) hospital admission, unscheduled contact physician, discomfort, impairment of activities, multiple source bleeding, hemoglobin drop > 3 g/dL. - HASBLED >3 - kidney of liver failure  Consider deprescribing heparins in case of high risk at bleeding: - uncontrolled hypertension (systolic pressure >179 mmHg) - uncontrolled bleeding - relevant recent spontaneous bleeding - clinically relevant bleeding: medical intervention or (prolonged) hospital admission, unscheduled contact physician, discomfort, impairment of activities, multiple source bleeding, hemoglobin drop > 3 g/dL. - HASBLED >3 - kidney of liver failure  Consider deprescribing Direct Oral Anticoagulants (DOAC) in case of high risk at bleeding: - uncontrolled hypertension (systolic pressure >179 mmHg) - uncontrolled bleeding - relevant recent spontaneous bleeding - clinically relevant bleeding: medical intervention or (prolonged) hospital admission, unscheduled contact physician, discomfort, impairment of activities, multiple source bleeding, hemoglobin drop > 3 g/dL. - HASBLED >3 - kidney of liver failure | | | | | 88,6%  100%  95,5%  100% | 1  0  1  1 | 0%  0%  0%  0% |  |
| Blood glucose-lowering medication | Fast-acting insulins  Long-acting insulins  Biguanides  Sulphonylureas  Dipeptyl peptidase 4 (DPP4) inhibitor  Glucagon-like Peptide-1 (GLP-1) analogues | | A10AB  A10AE  A10BA  A10BB  A10BH  A10BJ | Consider changing fast-acting insulins to a simpler regimen; using longer acting insulin in DM type 1 and 2 and deprescribing short or rapid acting insulins to few times a day in patients with few or unregular intake, deterioration of clinical condition  Consider lowering long-acting insulin if prescribed for DM type 1 and 2 with few or unregular intake, deterioration of clinical condition. OF note: in case of DM type 1: do not lower the dose to <10E/day  Consider deprescribing biguanides (i.e. metformin) if prescribed for mild hyperglycemia for secondary prevention of diabetic associated events  Consider deprescribing Sulphonylureas (i.e. glibenclamide /gliclazide/ tolbutamide/chlorpropamide) if prescribed for DM type 2 and prevention of diabetic associated events  Consider deprescribing Dipeptyl peptidase 4 (DPP-4) inhibitor (Linagliptin, saxagliptin, sitagliptin, vildagliptin) if prescribed for DM type 2 and secondary prevention of diabetic associated events  Consider deprescribing Glucacon-like Peptide-1 (GLP-1) analogues (Dulaglutide/exenatide/liraglutide/lixisenatide/semaglutide) if prescribed for DM type 2 and secondary prevention of diabetic associated events | | | | | 86,7%  90,9%  89,1%  91,3%  81,6%  94,3% | 1  1  1  1  1  1 | 2,2%  2,3%  0%  0%  0%  0% |  |
| Digestive system medication | H2-receptor antagonists  Proton Pump Inhibitor (PPI) | | A02BA  A02BC | Consider deprescribing H2-receptor antagonist if prescribed without a clear medical history of gastrointestinal bleeding, peptic ulcer, gastritis, gastro-esophageal reflux disease or use of NSAIDs and steroids  Consider deprescribing H2-receptor antagonist if prescribed for uncomplicated gastric ulcer/erosive peptic esophagitis more than 8 weeks ago and no persistent symptoms  Consider tapering and if possible stopping Proton Pump Inhibitor (PPI) if prescribed without a clear medical history of gastrointestinal bleeding, peptic ulcer, gastritis, gastro-esophageal reflux disease or use of NSAIDs and steroids | | | | | 91,5%  78,3%  88,6% | 0  1  1 | 0%  4,3%  0% |  |
| Osteoporosis medication | Drugs affecting bone structure and mineralization | | M05B | Consider deprescribing drugs affecting bone structure and mineralization (i.e. bisphosponates, denosumab) if prescribed for osteoporosis | | | | | 91,5% | 1 | 0% |  |
| Lower urinary tract medication | Urologicals | | G04 | Consider deprescribing urologicals (alpha adrenoreceptor antagonists and testosterone-5 alpha reductase inhibitors) if prescribed for lower urinary tract symptoms (LUTS) (e.g. urinary incontinence, nocturnal polyuria, benign prostate hypertrophy, long-term urinary catheter) and risk at falling | | | | | 86,4% | 0 | 2,3% |  |
| Miscellaneous medication | Supplements  Systemic estrogens | | A11+ A12  G03C | Consider deprescribing supplements (vitamins and mineral supplements) when prescribed for prophylaxis  Consider deprescribing systemic estrogens if not prescribed for moderate-severe climacteric complaints | | | | | 100%  82,9% | 0  1 | 0%  0% |  |
| **Life-expectancy: Three months or less** | |  |  |  | | | | |  |  |  |  |
| **Medication Class** | **Medication** | |  | **Recommendation** | | | | | **% Agreement** |  |  |  |
| Cardiovascular medication | ACE-inhibitor / Angiotensin II receptor blockers (C09)  Beta-blocking agent  Diuretics | | C09  C07  C03 | Consider deprescribing ACE-inhibitor / Angiotensin-II-receptor blocker if prescribed for mild-moderate hypertension (stage 1-2-3- hypertension, systolic blood pressure < 179, diastolic blood pressure < 110 mmHg i.e. stage 1-2-3 hypertension)  Consider tapering and if possible stopping beta blocking agents if prescribed for management of stable coronary artery disease (defined as history of angina pectoris in the presence of either risk factors for or known atherosclerotic cardiovascular disease)  Consider deprescribing diuretics if prescribed for mild-moderate hypertension(systolic blood pressure < 179, diastolic blood pressure < 110 mmHg i.e. stage 1-2-3 hypertension) | | | | | 89,4%  74,4%  78,8% | 1  1  1 | 0%  2,3%  0% |  |
| Miscellaneous | Anticholinesterase | | N07AA | Consider deprescribing anticholinesterase if prescribed for Alzheimer’s disease/dementia | | | | | 91,1% | 1 | 2,2% |  |
| **Life-expectancy: One month or less** | | | | |  |  |  |  |  |  |  |  |
| **Medication Class** | **Medication** | |  | **Recommendation** | | | | |  |  |  |  |
| Digestive system medication | Proton Pump Inhibitor (PPI) | | A02BC | Consider tapering and if possible stopping Proton Pump Inhibitor (PPI) if prescribed for uncomplicated gastric ulcer/erosive peptic esophagitis more than 8 weeks ago and no persistent symptoms | | | | | 81,8% | 1 | 2,3% |  |
